# The Iodoform, Intra-uterine Treatment during the Puerperium

**Published:** 1884-12

**Authors:** 


### EDITORIAL.

THE IODOFORM, INTRA-UTERINE TREATMENT DURING THE PUERPERIUM.

Dr. Emil Ehrendorfer has briefly sketched the iodoform, intra-uterine treatment, during the *puerperium*, in an interesting paper, appearing in the *Archiv für Gynækologie*, Bd. XXII., Heft I. The *Medical News* called attention to the Vienna method of the use of this agent. Vague allusions have, from time to time, appeared in other American medical journals, but the subject has excited little general interest. Certain it is, that the greater number of American obstetricians and gynecologists have no practical knowledge of this invaluable mode of therapy. We desire most earnestly to bring the subject before the profession.

Gynecologists, who believe in antiseptic midwifery at all, are prepared to accept the proposition, that irrigation of the uterine cavity with some antiseptic solution, irrespective of the reasons for operative interference, is indicated, after all procedures in which the *cavum uteri* has been invaded by the hand, or any instrument. In event of profuse ichorous secretion from the *endometrium*, irrigation of the uterine cavity, to be at all effective, must be frequently repeated. Frequent irrigation of the uterine cavity is contraindicated by the danger of trauma and sepsis, attendant upon repeated introductions of the uterine tube through the sensitive genital tract. Moreover, the plan is not feasible in private practice by reason of the time and attention required from the physician himself. Under such conditions, the introduction of an iodoform *bacillus* within the uterine cavity fulfills a vital indication. The local action of iodoform cannot be regarded as definitely settled at the present time.

Certain investigators believe that it has no action in the direct destruction of microbes and animal poisons. All investigators, however, unite in the opinion that it offers an incongenial soil for conditions favoring decomposition.

The quantity of iodoform, which can be safely placed within the uterine cavity, is six grammes. König and others have demonstrated that iodoform toxæmia is an excessively rare occurrence in doses under ten grammes.

Kowalski has observed the physiological action of iodoform in 326 cases of various diseases, and in his own case. He concludes that a daily dose of six grammes of pure iodoform has no toxic effect. If, however, in a concrete case, toxic symptoms appear, it is an exceedingly simple matter to wash out the remaining iodoform, and limit the absorption. The *bacilli* are elongated, tent-like bodies, with an ampulla, corresponding to the *fundus*.

The prescription is as follows:

℞ Iodoformi pulv.........................20.0
Gummi Arabici } ....................
Glycerini......... } ana............... 2.0
Amyli puri...... } ....................

F. bacilli nr. 3, longit. cm. 5–6.

In November, 1881, the iodoform, intra-uterine treatment during the *puerperium*, was instituted in the Lying-in wards of Carl Braun, Josef Spaeth, and Gastav Braun, Vienna General Hospital. The pathological conditions, under which the iodoform has been used, have been varied, and have presented every degree of severity. The verdict of three years' trial, in a fabulous number of cases, is emphatic commendation of this method of treatment.

